# AI-Driven Cybersecurity in IoT-Based Systems

**DOI:** 10.3390/s25237254

**Published:** 2025-11-28

**Authors:** Wenbing Zhao, Pan Wang

**Affiliations:** 1 Department of Electrical and Computer Engineering, Cleveland State University, Cleveland, OH 44115, USA; 2 School of Modern Posts, Nanjing University of Posts and Telecommunications, Nanjing 210023, China; wangpan@njupt.edu.cn

## 1. Introduction

With the rapid development of the Internet of Things (IoT) [1,2], including Industrial IoT and Home IoT, sensors and IoT devices are starting to play an increasingly important role in various fields such as smart grids, power plants, manufacturing, and supply chains. Such sensors and IoT-based systems could be vulnerable to various cyberattacks [3,4]. As an innovative data-driven paradigm, artificial intelligence (AI) has revolutionized several classical areas such as image, video, speech, and text processing [5,6,7]. However, when applying to mitigating cyberattacks, the inherent limitations of AI, such as data dependence, weak generalization, and lack of explainability, have obstructed the development of AI-driven cybersecurity [8,9]. Furthermore, sensors and IoT devices have imposed additional obstacles, including low power, heterogeneity, and weak computing power, which makes applying AI for cybersecurity even more challenging [10]. The aim of this Special Issue is to attract studies that have applied AI and other cutting-edge technologies to enable and empower cybersecurity in sensors and IoT-based systems.

## 2. Contributions

This Special Issue accepted five research articles and one comprehensive review. These studies focused on various issues related to cybersecurity in IoT-based systems, ranging from enhancing the interpretability of feature selection and introduction detection in IoT-based systems (1 contribution), detecting and mitigating attacks in the physical layer (2 contributions), and blockchain-facilitated cybersecurity in IoT-based systems (3 contributions).

### 2.1. Interpretable AI in Intrusion Detection

In contribution 1, Chen et al. performed feature selection based on the ranking of features using the SHAP method [11] with a deep learning model. SHAP is an explainability method based on Shapley values from cooperative game theory. SHAP treats prediction as a “payout” in a game where features are “players.” More specifically, SHAP calculates each feature’s contribution by averaging its marginal impact across all possible feature subsets. SHAP follows several key principles when calculating the value for each feature. First, it distributes the prediction difference fairly among features (i.e., fair attribution). Second, the sum of all feature contributions equals the model output, which ensures additive feature contributions. Third, if a model changes so a feature contributes more, its SHAP value will not decrease to ensure consistency.

In one dataset used for the study (CICIDS2017 [12]), the top 10, 15, and 20 features were selected from the 78 total features based on sorted SHAP values. For classification, the authors experimented with two different models, random forest (RF) and convolutional neural network (CNN). The results showed that the features selected by SHAP outperformed traditional feature selection methods when using the top 20 and 15 features. Furthermore, the accuracy actually improved when using the top 20 features compared with using the full 78 features. On the other hand, the results were mixed when using another dataset (NSL-KDD [13]). While the top 20 and 15 features selected via SHAP tied or outperformed traditional feature selection methods for CNN and RF, the accuracy was reduced compared with when using the full 38 features for CNN. For RF, using top 20 features obtained via SHAP outperformed using the full 38 features. However, the RF performance was noticeably worse than that of CNN.

### 2.2. Physical Layer Cybersecurity

In contribution 2, Qi, Liu, and Ye introduced an attention-enhanced defensive distillation network (AEDDN) to improve robustness and accuracy in vehicle-to-everything (V2X) millimeter-wave communication in the presence of adversarial attacks. The AEDDN model combines the transformer algorithm with defensive distillation. The goal is to leverage the transformer’s attention mechanism to focus on critical channel features and adapt to complex conditions. Defensive distillation is used to smooth the decision boundaries, which reduces the sensitivity to small perturbations.

In contribution 3, Senol et al. proposed a new method to identify tampered radio frequency transmissions by employing a suite of anomaly detection algorithms, including local outlier factor, isolation forest, variational autoencoder, traditional autoencoder, and principal component analysis. The context of the study is a LoRa-based IoT network [14]. LoRa refers to a long-range, low-power, and low-bandwidth wireless communication system. A key insight of the study is the employment of image-based tampered frequency techniques for securing LoRa transmissions.

### 2.3. Blockchain-Facilitated Cybersecurity

In contribution 4, Wang et al. proposed an extended robot operating system to protect the privacy of sensor data. The system utilizes blockchain [15] for data immutability protection of the data content identifiers, InterPlanetary File System (IPFS) [16] for data storage, as well as attribute-based hybrid encryption for fine-grained access control.

In contribution 5, Gao et al. also focused on privacy protection of the data using blockchain with an improved scheme for the distributed Oracle [17] data aggregation mechanism based on the Paillier encryption algorithm. In particular, the authors proposed an algorithm called PICA (Paillier-based InChain Aggregation), where the data are encrypted using the Paillier algorithm [18] and aggregated on the Chainlink blockchain [19]. Furthermore, random numbers are employed to mitigate the problem of dishonest Oracle machines.

In contribution 6, Zhao et al. provided a comprehensive review on blockchain-facilitated cybersecurity solutions for IoT devices in space–air–ground integrated networks (SAGIN) [20,21]. First, the authors identified the objectives and the context of the blockchain-based solutions for SAGIN. Second, the authors investigated how blockchain technology has been used to accomplish the objectives. Deep technical details on how blockchain has been used in this specific field were presented. Third, the authors provided a critique on the technical correctness of the blockchain-based solutions and pointed out serious technical issues in the proposed solutions. The most pervasive misconception is that higher-level trustworthiness can be achieved by using any form of blockchain, which is wrong, as elaborated in [22]. Finally, the authors proposed a guideline on when the blockchain technology can be useful for IoT and SAGIN and what types of blockchain may be useful to enhance the security of ubiquitous IoT in SAGIN.
